# Blood Flow Restriction Exercise Attenuates the Exercise-Induced Endothelial Progenitor Cell Response in Healthy, Young Men

**DOI:** 10.3389/fphys.2019.00447

**Published:** 2019-04-17

**Authors:** Ryan Montgomery, Allan Paterson, Chris Williamson, Geraint Florida-James, Mark Daniel Ross

**Affiliations:** School of Applied Sciences, Edinburgh Napier University, Edinburgh, United Kingdom

**Keywords:** endothelial progenitors, exercise, endothelial, angiogenesis, blood flow restricted exercise

## Abstract

Endothelial progenitor cells (EPCs) are a vasculogenic subset of progenitors, which play a key role in maintenance of endothelial integrity. These cells are exercise-responsive, and thus exercise may play a key role in vascular repair and maintenance via mobilization of such cells. Blood flow restriction exercise, due to the augmentation of local tissue hypoxia, may promote exercise-induced EPC mobilization. Nine, healthy, young (18–30 years) males participated in the study. Participants undertook 2 trials of single leg knee extensor (KE) exercise, at 60% of thigh occlusion pressure (4 sets at 30% maximal torque) (blood flow restriction; BFR) or non- blood flow restriction (non-BFR), in a fasted state. Blood was taken prior, immediately after, and 30 min after exercise. Blood was used for the quantification of hematopoietic progenitor cells (HPCs: CD34^+^CD45^dim^), EPCs (CD34^+^VEGFR2^+^/CD34^+^CD45^dim^VEGFR2^+^) by flow cytometry. Our results show that unilateral KE exercise did not affect circulating HPC levels (*p* = 0.856), but did result in increases in both CD34^+^VEGFR2^+^ and CD34^+^CD45^dim^VEGFR2^+^ EPCs, but only in the non-BFR trial (CD34^+^VEGFR2^+^: 269 ± 42 cells mL^-1^ to 573 ± 90 cells mL^-1^, pre- to immediately post-exercise, *p* = 0.008; CD34^+^CD45^dim^VEGFR2^+^: 129 ± 21 cells mL^-1^ to 313 ± 103 cells mL^-1^, pre- to 30 min post-exercise, *p* = 0.010). In conclusion, low load BFR exercise did not result in significant circulating changes in EPCs in the post-exercise recovery period and may impair exercise-induced EPC mobilization compared to non-BFR exercise.

## Introduction

Endothelial progenitor cells (EPCs) were first discovered in 1997 by Asahara et al. (1997). These peripheral blood mononuclear cells (PBMC) could form endothelial cell-like networks and differentiate into mature endothelial cell phenotypes *in vitro*. These cells are bone marrow-derived and can be mobilized in response to vascular injury or an inflammatory stimulus (Asahara et al., 1999; Shintani et al., 2001). Since 1997, there have been a plethora of studies reporting their vasculogenic, angiogenic and vascular repair properties (Abd El Aziz et al., 2015; Yu et al., 2016; Chilla et al., 2018; Kong et al., 2018). In vascular disease states and in advancing age, circulating EPC number and function are lower (Fadini et al., 2006; Thijssen et al., 2006; Xia et al., 2012;Liao et al., 2014). It is reported that these cells are independent predictors of endothelial function (Sibal et al., 2009; Bruyndonckx et al., 2014), and may also be predictors of cardiovascular mortality (Rigato et al., 2016).

Exercise has been shown to improve endothelial function (Black et al., 2009), which is likely due to the regular elevations in shear stress (Tinken et al., 2010) that occurs due to elevated cardiac output and metabolic demand of the working muscle. Recently, acute bouts of exercise have been shown to mobilize EPCs from bone marrow and into the circulation (Van Craenenbroeck et al., 2008; Ross et al., 2014), which may contribute to endothelial growth and repair. However, in some populations, such as older individuals (Ross et al., 2018) and heart failure patients (Van Craenenbroeck et al., 2011), the acute exercise response is impaired.

Blood flow restriction (BFR) exercise has been recently used to augment muscle hypertrophy (building muscle tissue) and strength whilst undertaking low-load resistance training (Abe et al., 2012). This is of interest to individuals who are unable to undertake higher-load training, such as injured athletes, older or diseased populations. Interestingly, BFR exercise may improve vascular function compared to non-restricted exercise (matched for workload) (Horiuchi and Okita, 2012), which may make BFR exercise an option for individuals with vascular disease who cannot undertake moderate-to-high intensity exercise. One potential mechanism is the exercise-induced elevations in key angiogenic stimuli, such as vascular endothelial growth factor (VEGF), which is elevated in low-load BFR exercise compared to low-load exercise without BFR as a control (Larkin et al., 2012; Ferguson et al., 2018). This, in addition with other hypoxic stimuli, may stimulate the mobilization and recruitment of EPCs from the bone marrow, which can then act to stimulate vascular repair in areas of endothelial damage/dysfunction. Therefore we wanted to investigate the influence of BFR exercise on EPC mobilization in young, healthy men. It was hypothesized that BFR exercise would augment the exercise-induced mobilization of EPCs.

## Materials and Methods

### Ethics Statement

This study was carried out in accordance with the recommendations of Edinburgh Napier University Research and Ethics Governance Committee. The study was ethically approved by Edinburgh Napier University Research and Ethics Governance Committee. All participants gave written informed consent in accordance with the Declaration of Helsinki.

### Participants

Nine healthy adult males (age 18–30 years) volunteered to take part in the study. Participants were physically active (took part in formal exercise training at least 2×per week), non-obese (BMI < 30 m⋅kg^2^), non-smokers, and not taking any medications. Participants were told to refrain from undertaking strenuous exercise for 2 days prior to the visits to the Human Performance Laboratory. Participant characteristics are provided in Table 1.

**Table 1:** Participant characteristics (*n* = 9).

Characteristics	
Age (years)	21 ± 1
Body Mass Index (m⋅kg^2^)	25.77 ± 1.10
Systolic Blood Pressure (mmHg)	131 ± 2
Diastolic Blood Pressure (mmHg)	78 ± 2
Knee Extensor Maximal Torque (N)	255 ± 16
30% Maximal Torque (N)	75 ± 5


*Values shown are mean ± SEM.*

### Experimental Design

In a repeated measures randomized design, participants performed fasted, unilateral, low-load, knee extension (KE) exercise (dominant leg) on an isokinetic dynamometer (Cybex Humac Norm, Computer Sports Medicine Inc., United States). Two experimental trials were undertaken, a low-load KE exercise (1) with and (2) without BFR, with a minimum of 1 week apart.

### Assessment of Peak Torque

One week prior to the first experimental trial, participants undertook a KE maximal torque test (1RM) on the isokinetic dynamometer after a 5 min warm up on a bicycle ergometer (75 W, 60 rpm). Participants initially performed 5 repetitions, through 90° range of motion at 60° per second concentrically at ∼75% of maximal effort, followed by a short rest period before attempting a further 5 repetitions, with participants given the instruction to produce maximal efforts. 1RM was determined as the maximal voluntary torque produced throughout a controlled and full range of motion repetition. After the maximal torque assessment, participants were fitted with the pneumatic cuff placed on the dominant thigh, and performed 5 repetitions to familiarize the participants with the BFR prior to the experimental trials.

### Experimental Trials

After a minimum of 7 days following the maximal torque assessment, participants returned to the Human Performance Laboratory in a fasted state, having refrained from strenuous exercise for 48 h prior to the visit, and having refrained from caffeine and alcohol the night before the visit. Participants underwent a warm up consisting of a 5 min cycle (Monark 824E, Monark Exercise AB, Sweden) at 75 W at 60 rpm, followed by 5 warm up KE repetitions at 20% 1RM. The exercise trial consisted of 4 sets of unilateral knee extensions at 20% 1RM at a cadence of 1.5 s per contraction phase across 90° range of motion and at a speed of 60° per second (1 set of 30 repetitions, followed by 3 sets of 15 repetitions) interspersed with 30 s recovery periods, similar to previous work in this area (Drummond et al., 2008; Ferguson et al., 2018). Throughout the 4 sets, participants were fitted with a thigh occlusion cuff (Hokanson CC17 Thigh Cuff, Hokanson Inc., United States) at the most proximal end of their dominant leg, either inflated to 60% of their thigh occlusion pressure (BFR) or 5 mmHg (non BFR). Thigh occlusion pressure was identified as the highest pressure at which arterial blood flow could not be detected by a vascular Doppler (BT-200 Vascular Doppler, Bistos Co., Ltd., South Korea) on the posterior tibial artery. Occlusion pressure was maintained for the entirety of the exercise bout including inter-set rest periods. Blood samples were taken pre-, immediately post- and 30 min post-exercise by venepuncture (see section “Blood Sampling and EPC Phenotyping”).

All participants undertook the exercise at the same time of day as their first experimental trial (0830-1000).

### Blood Sampling and Endothelial Progenitor Cell Phenotyping

Blood was taken from participants before, immediately post- and 30 min post-exercise bout by a trained phlebotomist using a 21-guage needle (BD Luer-Lok^TM^, BD Biosciences, United Kingdom). Peripheral blood from the antecubital vein was drawn into 2 × 6 mL vacutainers spray-coated with EDTA anti-coagulant (BD Biosciences, United Kingdom), with the first 3 mL discarded to avoid contamination of circulating endothelial cells produced with the initial venepuncture. Differential leukocyte counts were determined using semi-automated hematology analyser (XS 1000i, Sysmex, United Kingdom).

**FIGURE 1:**
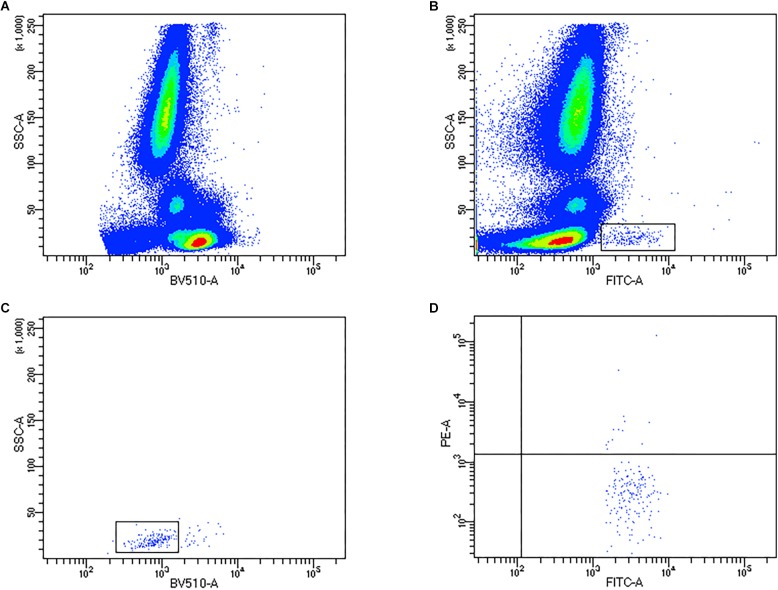
Representative flow cytometry density and dot plots to quantify endothelial progenitor cells (EPCs). **(A)** Identification of CD45^+^ PBMCs, **(B)** CD34^+^ gating, **(C)** CD45^dim^ expression on CD34^+^ progenitors, **(D)** co-expression of VEGFR2.

For flow cytometric quantification of EPCs, briefly, 200 μL of whole blood was incubated with 5 μL of anti-CD34 FITC, 5 μL anti-CD45 BV510, and 10 μL anti-VEGFR2 PE (all BD Biosciences, United Kingdom) for 30 min away from light, followed by the addition of 2 mL Lysis (BD Pharm Lyse^TM^, BD Biosciences, United Kingdom) prior to flow cytometric analysis. EPCs were quantified using a BD FACS Celesta (BD Biosciences, United Kingdom) flow cytometer, equipped with a Violet laser (405 nm), Blue laser (488 nm) and a Yellow-Green laser (561 nm). Compensation was performed prior to the study to correct for any spectral overlap, and controls (fluorescence minus 1) were used for each participants’ visit. Circulating EPC data was obtained using BD FACS Diva (BD Biosciences, United Kingdom). Firstly, CD45^+^ PBMCs were gated (Figure 1A), followed by identification of SSC-low and CD34^+^ events (Figure 1B), subsequent low expression of CD45 (CD45dim; Figure 1C) and VEGFR2^+^ events (Figure 1D) were identified. A minimum of 250,000 CD45^+^ PBMC events were collected per sample. Circulating concentrations of progenitor cells were obtained using a dual platform method, by multiplying the percentage values obtained from the flow cytometer by the corresponding leukocyte count as obtained from hematology analysis.

Changes in blood volume was accounted for by using known measures of hematocrit and hemoglobin obtained from automated hematology analysis (Sysmex, XS 1000i, United Kingdom) (Dill and Costill, 1974).

### Statistical Analysis

All data are presented as mean ± SEM unless otherwise stated. Two-way analyses of variance (ANOVA) with repeated measures were performed to investigate main effects of the exercise bout on circulating progenitor cells, and interaction of time (pre-, post-, 30 min post-exercise) × trial (BFR vs. non-BFR). When significant differences were detected, Bonferroni *post hoc* tests were performed to determine location of the effect (pre, post- 1 h post-exercise). Effect sizes are presented as Pearson’s *r* coefficient for ANOVA analyses, and Cohen’s *d* for paired analyses. Data was analyzed using GraphPad Prism 8 for Windows (GraphPad Software Inc., United States). Significance alpha was set at *p* < 0.05.

## Results

### Unilateral Knee Extension Exercise Performance

There was no difference in torque produced during either BFR or non-BFR trial (75.89 ± 4.86N vs. 76.76 ± 5.96N, *p* = 0.911), which equated to 29.77 ± 1.12% vs. 31.44 ± 1.77% of maximal torque (*p* = 0.423).

### Immunological Responses

There was no main effect of the exercise bout on circulating neutrophils [*F*_(2,48)_ = 0.383, *p* = 0.684, *r* = 0.09] or monocytes [*F*_(2,48)_ = 1.613, *p* = 0.210, *r* = 0.18] in the trials, but there was a main effect of exercise (pre- to post- and 30 min post-exercise) on circulating lymphocytes [*F*_(2,48)_ = 13.45, *p* < 0.001, *r* = 0.47]. However, for all three subsets of circulating leukocytes, there was no time × trial interaction (*p* > 0.05). Leukocyte changes in response to BFR and non-BFR exercise are shown in Table 2.

**Table 2:** Circulating leukocyte changes in response to blood flow restricted (BFR) and non- Restricted (non-BFR) Exercise (*n* = 9).

		Pre	Immediately Post-	30 min Post-	Main effect of exercise	Time x Trial Interaction
Neutrophils (cells × 10^9⋅^L^-1^)	BFR	3.95 ± 0.44	4.47 ± 0.62	4.37 ± 0.59	*F*_(2,48)_ = 0.38	*F*_(2,48)_ = 0.13
	Non-BFR	3.38 ± 0.51	3.46 ± 0.54	3.93 ± 0.63	3, *p* = 0.684	7, *p* = 0.872
Monocytes (cells × 10^9⋅^L^-1^)	BFR	0.56 ± 0.06	0.69 ± 0.08	0.56 ± 0.04	*F*_(2,48)_ = 1.61	*F*_(2,48)_ = 0.51
	Non-BFR	0.53 ± 0.04	0.57 ± 0.05	0.53 ± 0.05	3, *p* = 0.210	5, *p* = 0.601
Lymphocytes (cells × 10^9⋅^L^-1^)	BFR	1.83 ± 0.18	2.31 ± 0.15	1.54 ± 0.12	*F*_(2,48)_ = 13.45	*F*_(2,48)_ = 0.98
	Non-BFR	1.95 ± 0.09	2.31 ± 0.15	1.51 ± 0.05	0, *p* < 0.001^∗^	1, *p* = 0.382


*Values shown are mean ± SEM, ^∗^*p* < 0.001.*

### Endothelial Progenitor Cell Responses

There was no main effect of the exercise bout on CD34^+^ progenitor cells [*F*_(2,48)_ = 0.1559, *p* = 0.856, *r* = 0.06] or any interaction of time × trial [*F*_(2,48)_ = 0.2015, *p* = 0.818, *r* = 0.06]. There was a main effect of time on CD34^+^VEGFR2^+^ EPCs [*F*_(2,48)_ = 4.175, *p* = 0.021, *r* = 0.28]. However, as with CD34^+^ progenitors, there was no time × trial interaction [*F*_(2,48)_ = 1.199, *p* = 0.310, *r* = 0.16]. Likewise, there was a significant main effect of the exercise bout on CD34^+^CD45^dim^VEGFR2^+^ EPCs [*F*_(2,48)_ = 3.115, *p* = 0.049, *r* = 0.25], but no significant time × trial interaction was observed [*F*_(2,48)_ = 0.702, *p* = 0.501, *r* = 0.12].

**FIGURE 2:**
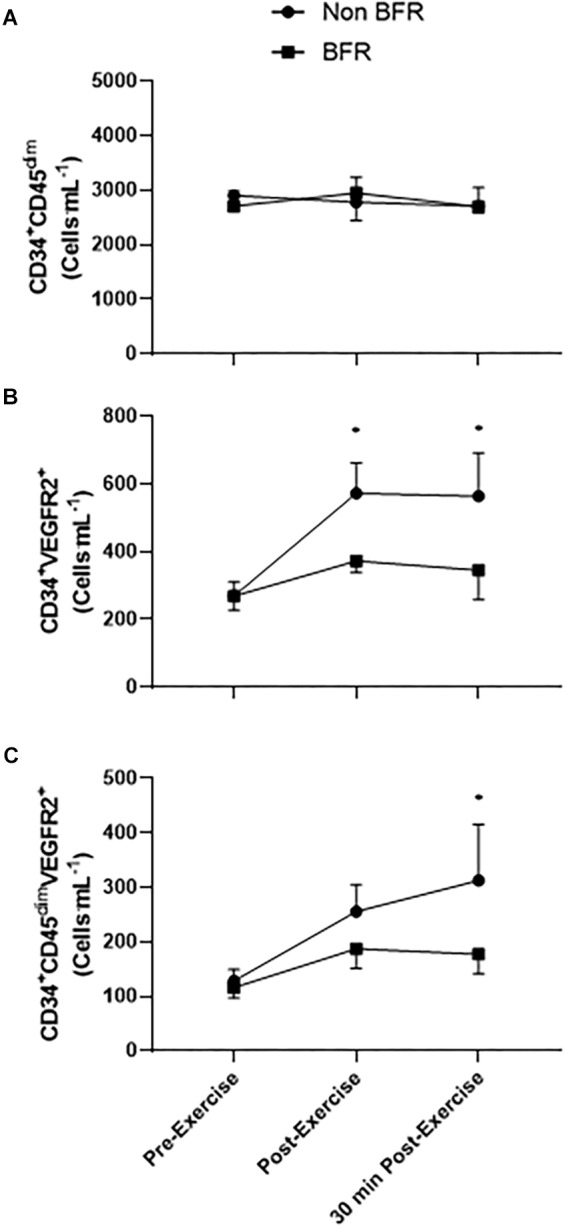
Circulating CD34^+^CD45^dim^, CD34^+^VEGFR2^+^ EPCs, and CD34^+^CD45^dim^VEGFR2^+^ EPCs in response to blood flow restricted (BFR) and non-restricted (non-BFR) exercise (*n* = 9). Values shown are mean ± SEM, ^∗^*p* < 0.05 vs. pre-exercise non-BFR only.

CD34^+^VEGFR2^+^ cells increased significantly from 269 ± 42 cells mL^-1^ at rest to 573 ± 90 cells mL^-1^ (*p* = 0.008, *d* = 1.37) immediately post-non-BFR exercise, with a non-significant increase of 269 ± 42 cells mL^-1^ to 373 ± 33 cells mL^-1^ in the BFR trial (*p* = 0.352, *d* = 0.87). CD34^+^VEGFR2^+^ EPCs were still significantly elevated 30 min post-exercise compared to pre-exercise levels, in the non-BFR trial only (269 ± 42 cells mL^-1^ to 564 ± 128 cells mL^-1^, *p* = 0.010, *d* = 0.98). CD34^+^CD45^dim^VEGFR2^+^ EPCs only significantly increased from pre- to 30 min post-exercise in the non-BFR trial (129 ± 21 cells mL^-1^ to 313 ± 103 cells mL^-1^, *p* = 0.010, *d* = 1.23), with no such statistical differences in the BFR trial (116 ± 19 cells mL^-1^ to 177 ± 35 cells mL^-1^, *p* = 0.010, *d* = 0.68) (Figure 2).

## Discussion

This is the first study to investigate the effect of an acute bout of BFR exercise on circulating progenitor cells. Our main finding of the study was that BFR exercise mitigated the increase in circulating CD34^+^VEGFR2^+^ and CD34^+^CD45^dim^VEGFR2^+^ EPCs shown in the non-BFR exercise trial. There was no statistical significant time × trial interaction, we found that only the non-BFR exercise resulted in a statistical significant increase in both CD34^+^VEGFR2^+^ and CD34^+^CD45^dim^VEGFR2^+^ cells in the circulation, both of which resulted in a large effect (Cohen’s *d* > 0.8). We did not observe any changes in either trial in total CD34^+^CD45^dim^ progenitor cells, suggestive of a specific exercise responsiveness of EPCs. We hypothesized that the BFR trial would augment the circulating EPC response to exercise, potentially due to elevation in local and systemic hypoxic and angiogenic stimuli that have been shown with acute bouts of BFR exercise (Larkin et al., 2012; Ferguson et al., 2018).

Our previous work and others have shown that exercise can stimulate the mobilization of EPCs from the bone marrow of healthy young and older adults (Van Craenenbroeck et al., 2008; Ross et al., 2014, 2018). These increases in EPCs are observed concomitantly with elevations in plasma VEGF levels (Adams et al., 2004; Möbius-Winkler et al., 2009; Ross et al., 2014, 2018). Interestingly, despite elevations in VEGF mRNA, the resulting plasma VEGF concentrations did not differ between BFR and non-BFR trials in a previous BFR study (Larkin et al., 2012). Work by Ferguson et al. (2018) observed that VEGF gene expression in skeletal muscle increased in BFR exercise more so than non-BFR exercise after 2 and 4 h. It is possible that hypoxic stimulus created by the BFR exercise, may result in sustained elevation in VEGF gene expression, which may result in increased skeletal muscle VEGF protein content and subsequent elevations in VEGF released into interstitial space and plasma after 4 h. Our study focused on the initial circulating EPC response to the exercise bout, and found that BFR exercise appears to blunt the EPC mobilization immediately post-exercise, and in the short term recovery period. However, there was a moderate-to-large effect for EPC mobilization post-BFR exercise (Cohen’s *d* between 0.67 and 0.87), however, this was still a lower effect than observed for non-BFR exercise. Future studies should employ further time points for analysis of EPC levels due to the possibility of any delayed VEGF release having a direct impact on EPC mobilization from the bone marrow.

Participants in the current study undertook a single leg KE exercise (of the dominant leg). Previous studies have employed BFR exercise in a bilateral exercise trial (Ferguson et al., 2018), or at a higher exercise intensity than our own (Larkin et al., 2012). We decided on a unilateral exercise trial and ∼30% of maximal torque from pilot testing for participants being able to withstand the exercise trial, however, we know that exercise intensity plays an important role in progenitor cell responses to exercise (Laufs et al., 2005), and likely that more muscle mass involved in exercise may stimulate a greater systemic response. Therefore we recommend that further studies are employed to ascertain role of exercise intensity, as well as occlusion pressure, on EPC kinetics in individuals to fully explore this area of study.

In addition to progenitor cell data, we also were able to quantify immunological response to the exercise trials. Either trial failed to stimulate significant changes in both neutrophils or monocytes. However, there was an effect of exercise on lymphocytes, with a significant redeployment of cells into the peripheral blood compartment, but there was no exercise × trial interaction. Behringer et al. (2018) observed significant elevations in absolute neutrophil count after 4 sets of BFR exercise (repetitions at 75% 1RM). Immunological responses to exercise are highly intensity-dependent (Rowbottom and Green, 2000), and therefore the difference in intensity between our 2 studies are likely to be the reason for the differences in our findings. However, our BFR trial (4 sets at ∼30% maximum torque) resulted in minimal immunological changes, and therefore may not perturb our immune system to the same extent as high intensity BFR exercise, thus making it an acceptable exercise mode for at-risk populations. However, more study is needed to investigate the influence of such bouts of exercise on specific immune cell subsets, such as T-cells, B-cells, NK-cells, and pro-inflammatory monocytes.

## Limitations

Our study has several limitations which must be appreciated. Firstly, our timescale of obtaining blood samples was limited, from pre-exercise to 30 min post-exercise. We observe a delayed angiogenic gene expression in response to BFR exercise, and thus, EPC response may also be delayed, on the basis that VEGF may stimulate exercise-induced EPC mobilization. In addition, our unilateral exercise protocol may not have been a sufficient stimulus for EPC mobilization. Despite this, we did observe a significant effect of low-intensity (∼30% maximal torque) unilateral KE exercise on EPCs in the non-restricted trial, suggestive of other factors at play other than VEGF or other angiogenic signaling proteins.

Our sample size (*n* = 9), was less than was targeted (*n* > 10 for power >95%) according to G^∗^power calculations. However, we achieved 92% power with the *n* = 9, and as such we are confident in our analyses of the data provided, which include larger effect sizes for changes in EPCs from pre-to-post-exercise in the non-BFR trial (Cohen’s *d* between 0.98 and 1.37) than the BFR trial (Cohen’s *d* between 0.67 and 0.87), which failed to statistically alter the levels of EPCs in peripheral blood of the participants.

## Conclusion

In summary, this is the first study to show that BFR exercise did not augment EPC response to exercise, and in fact blunted the EPC response to low load unilateral KE exercise in young, healthy males.

## Ethics Statement

This study was carried out in accordance with the recommendations of Edinburgh Napier University Research and Ethics Governance Committee. The study was ethically approved by Edinburgh Napier University Research and Ethics Governance Committee. All participants gave written informed consent in accordance with the Declaration of Helsinki.

## Author Contributions

MR, RM, AP, CW, GF-J designed the study. MR, RM, AP, and CW undertook the data collection. MR and RM analyzed the data. MR, GF-J wrote the manuscript. MR, RM, AP, CW, and GF-J reviewed the data and the manuscript. All authors read and approved of the manuscript.

## Conflict of Interest Statement

The authors declare that the research was conducted in the absence of any commercial or financial relationships that could be construed as a potential conflict of interest.
